# Supplementary material for: Cerium-Doped Iron Oxide Nanorod Arrays for Photoelectrochemical Water Splitting
Source: Molecules. 2022 Dec 19;27(24):9050. doi: 10.3390/molecules27249050 (PMC9780861; doi:10.3390/molecules27249050)
Supplement: Supplementary file 1 [file molecules-27-09050-s001.zip › molecules-2089978-supplementary.pdf]

Supplementary Information

Cerium-doped iron oxide nanorod arrays for photoelectrochemical water splitting

Hai-Peng Zhao [a,b,c], Mei-Ling Zhu [b,c,d], Hao-Yan Shi [b,c,e], Qian-Qian Zhuo [b,c], Rui Chen [b,c], Shi-Wei Lin [b,c], Mei-Hong Tong [b,c], Ming-Hao Ji [b,c], Xia Jiang [b,c], Chen-Xing Liao [a], Yan-Xin Chen [b,c,e,f*], Can-Zhong Lu [b,c,d,e,f*]

[a] *School of Rare Earth, Jiangxi University of Science and Technology, Ganzhou 341000, Jiangxi, China*

[b] *CAS Key Laboratory of Design and Assembly of Functional Nanostructures, and Fujian Provincial Key Laboratory of Nanomaterials, Fujian Institute of Research on the Structure of Matter, Chinese Academy of Sciences, Fuzhou 350002, China*

[c] *Xiamen Key Laboratory of Rare Earth Photoelectric Functional Materials, Xiamen Institute of Rare-earth Materials, Haixi Institutes, Chinese Academy of Sciences, Xiamen 361021, China*

[d] *School of Rare Earths, University of Science and Technology of China, Hefei 230026, China*

[e] *College of Chemical Science, University of Chinese Academy of Sciences, Beijing 100049, China*

[f] *Fujian Science & Technology Innovation Laboratory for Optoelectronic Information of China, Fuzhou 350108, China*

***Email:** yanxinchen@fjirsm.ac.cn and czlu@fjirsm.ac.cn

Table S1. H_2 Production of Ce-doped Materials

Materials	H_2 Production Of Materials	Ce-doped Materials	H_2 Production of Ce-doped Materials	Bias	Electrolyte	ref
Fe_2O_3 nanorods	3.96μmol $h^{-1}cm^{-2}$	Ce-Fe_2O_3 nanorods	122μmol $h^{-1}cm^{-2}$	1.6V *vs.* RHE	1MKOH	This work
ZnO nanowire	125ppm	CeO_2/Ce-ZnO nanowire	225ppm	1.0 V vs. RHE	0.1 MKOH with 0.1 M glycerol	[1]
$BaTiO_3$ nanoassemblies	13μmol $h^{-1}cm^{-2}$	Ce-$BaTiO_3$ nanoassemblies	23μmol h^{-1} cm^{-2}	0.7 V *vs.* Pt	1 mM NaOH	[2]
TiO2 nanotube	125ml $h^{-1}cm^{-2}$	Ce/Ce2O/CeO2/TiO2 nanotube	223μmol $h^{-1}cm^{-2}$	0.7 V vs. open circuit potential	0.1M Na2SO4 with 10vol % Ethylene glycol	[3]

Table S2. ICP and ICP-MS of Fe_2O_3 (FT) and Ce-doped Fe_2O_3 (1CFT, 5CFT, 10CFT).

Sample	Fe(ppb)	Ti(ppb)	atomic ratio(%)	Ce(ppb)	atomic ratio(%)
FT	5695	22.186	0.390%	0.0007	0.000012%
1CFT	4443	26.925	0.606%	0.368	0.0083%
5CFT	4521	40.967	0.906%	0.162	0.0036%
10CFT	4124	22.054	0.535%	0.115	0.0028%

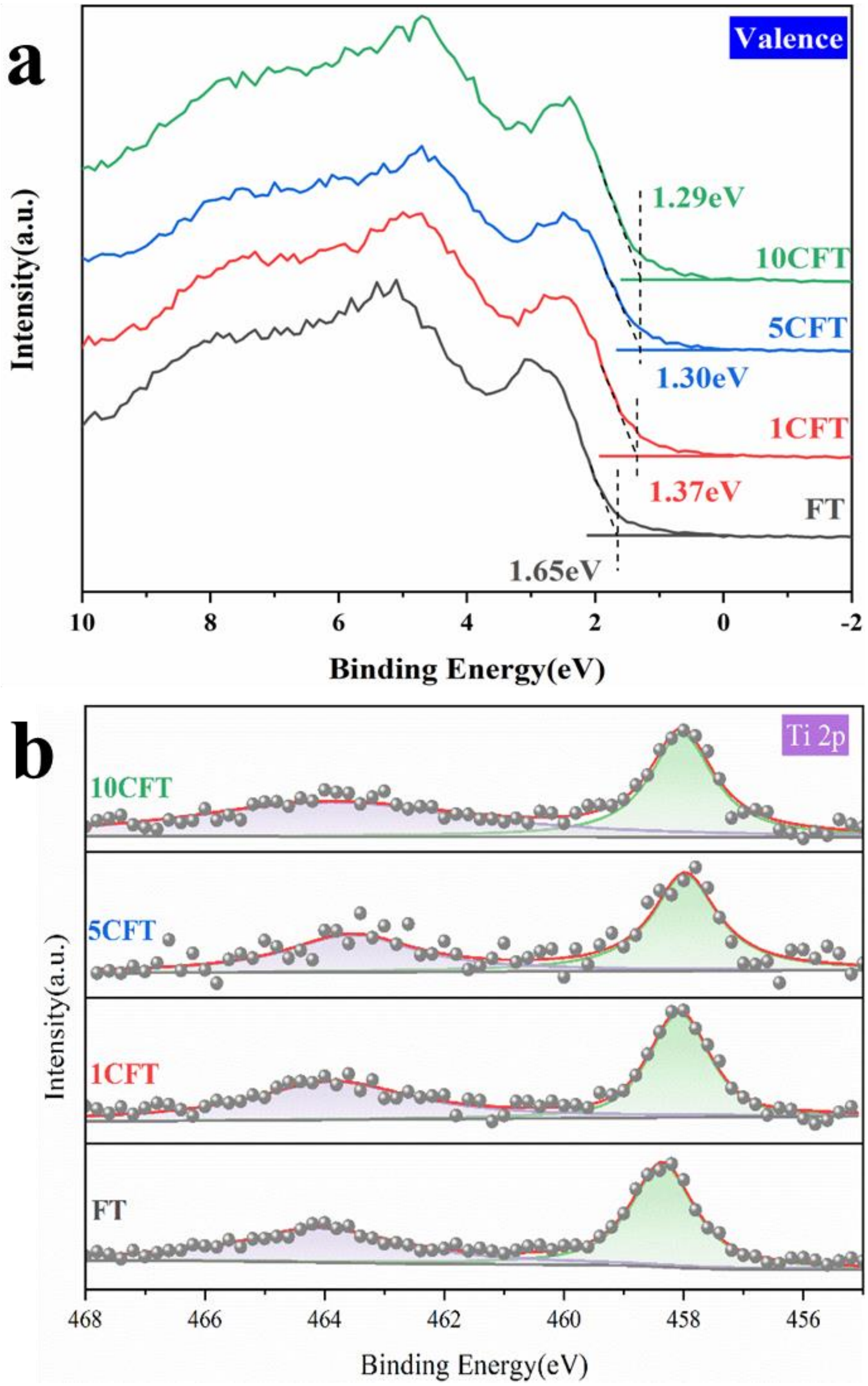

Figure S1. XPS Valence of Fe_2O_3 (FT) and Ce-doped Fe_2O_3 (1CFT, 5CFT, 10CFT).(a); Ti 2p (b)

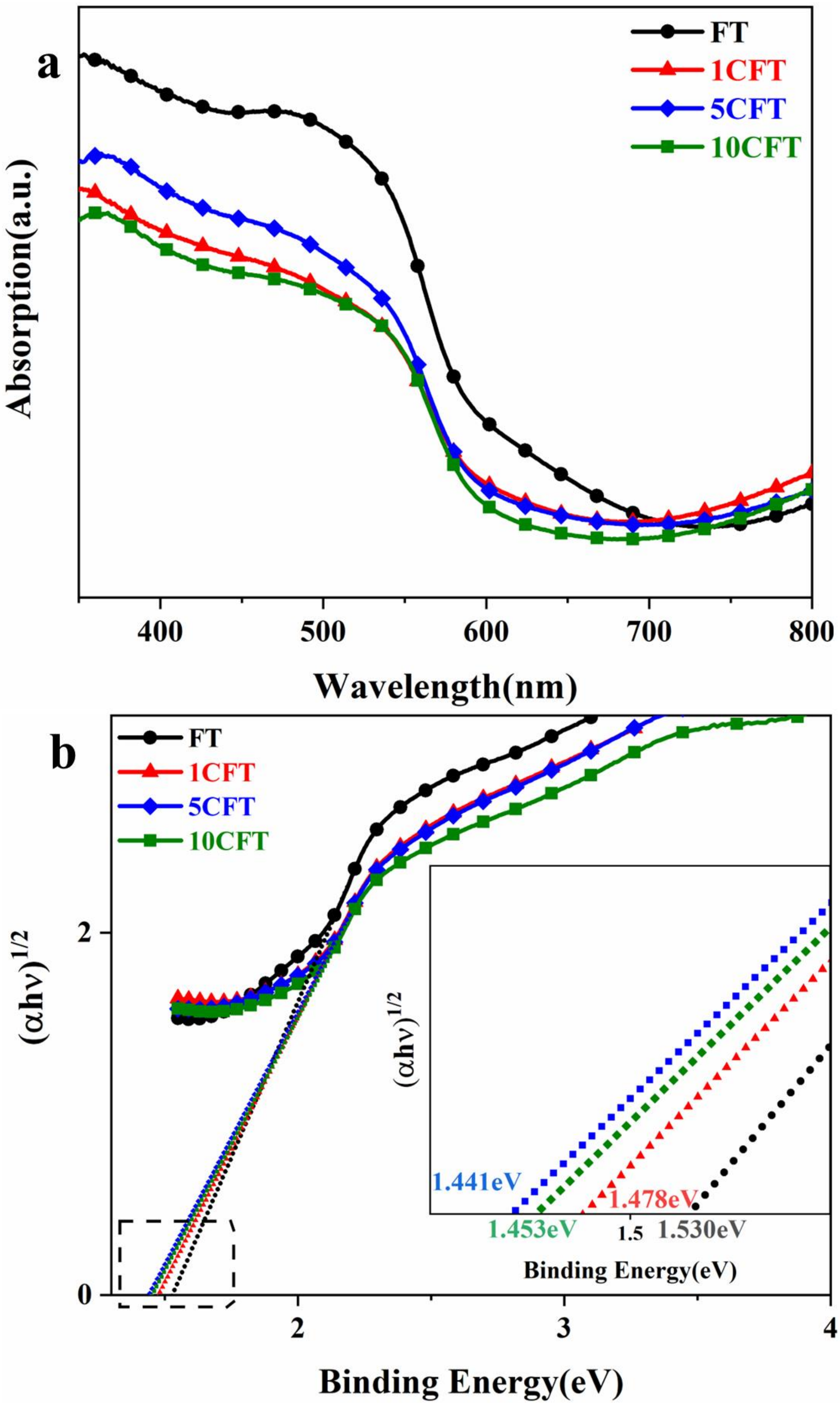

Figure S2. UV-vis absorption profile of Fe_2O_3 (FT) and Ce-doped Fe_2O_3 (1CFT, 5CFT, 10CFT). (a) and optical bandgaps, inset is the refined spectrum of 0.4eV-1.7eV(b)

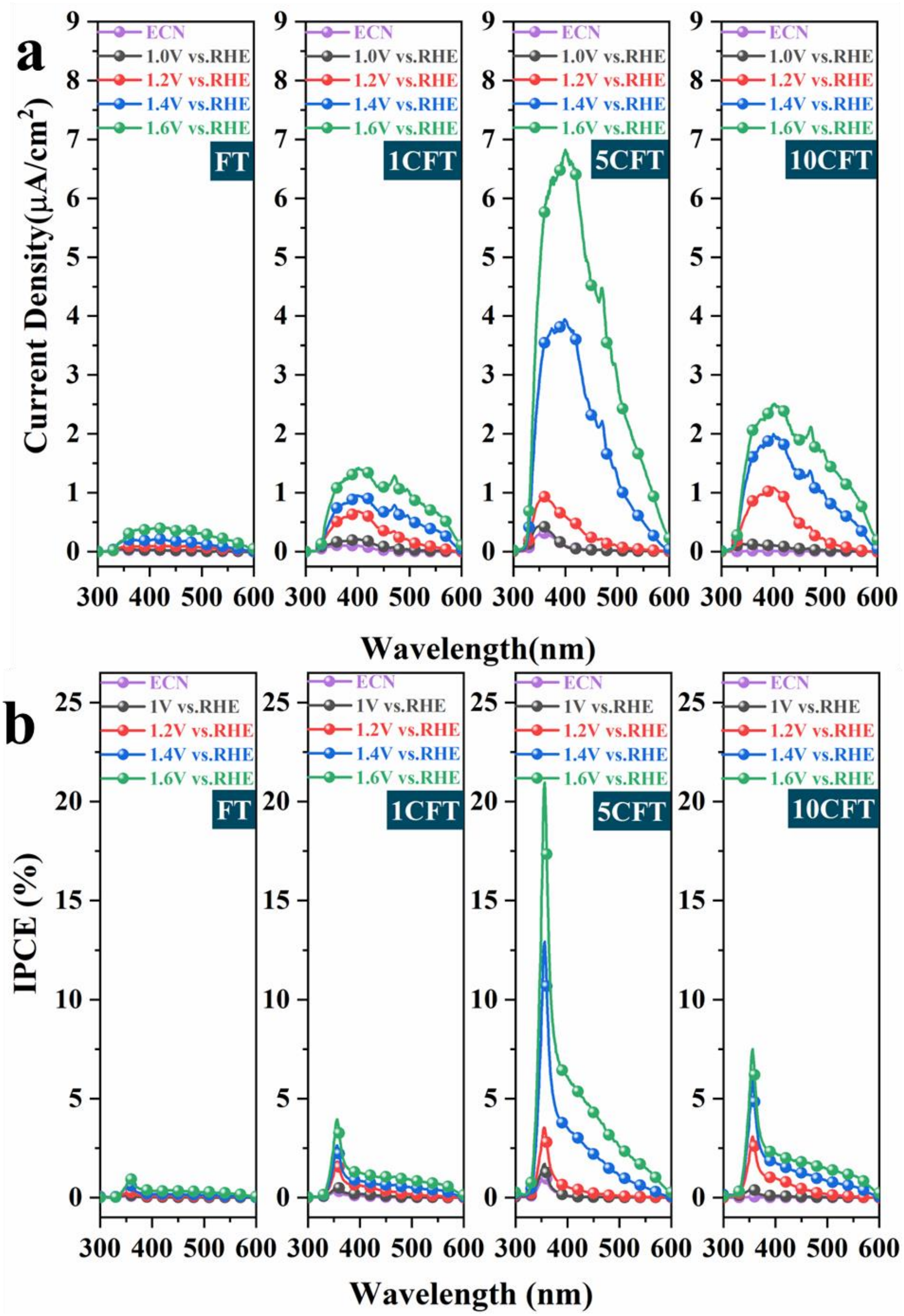

Figure S3. (a) Photocurrent density versus the monochromatic light; (b) IPCE values of FT, 1CFT, 5CFT, 10CFT under different bias

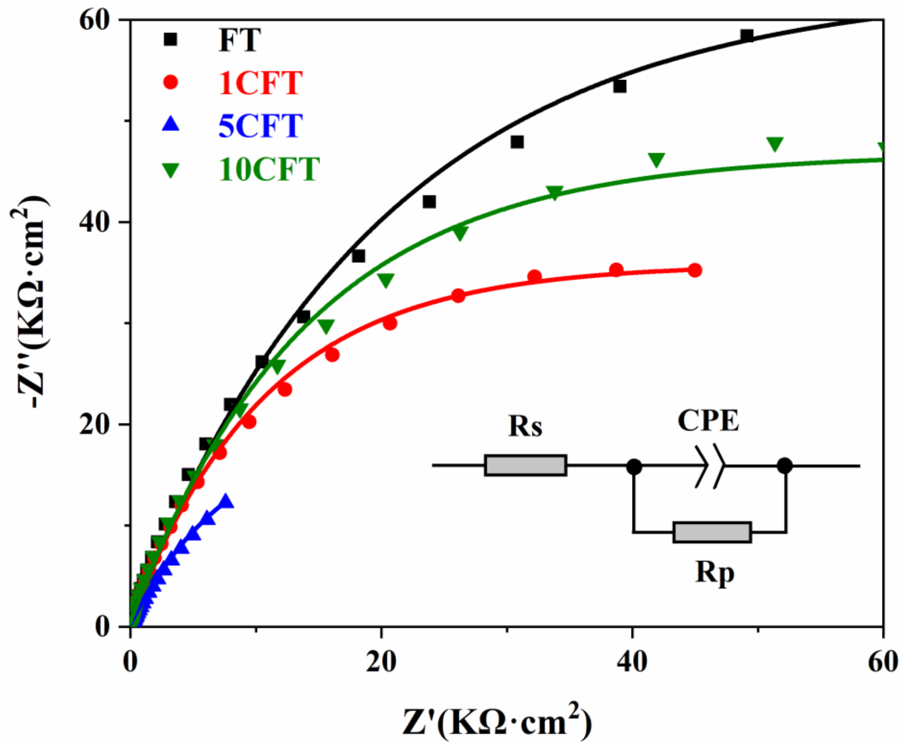

Figure S4 Electrochemical impedance spectroscopy (EIS) and Equivalent Circuit of Fe_2O_3 nanorods and Ce doped Fe_2O_3 nanorods samples

Table S3 Fitting data for each component in the fitted circuit for each sample

samples	Rs	CPE-σ	CPE-m	Rp
FT	2.0722	2.1915×10^{-5}	0.88228	1.6133×10^{5}
1CFT	1.2783	2.7865×10^{-5}	0.85492	0.89992×10^{5}
5CFT	1.1421	15.332×10^{-5}	0.75145	0.79914×10^{5}
10CFT	1.877	1.4352×10^{-5}	0.87341	1.0574×10^{5}

Rp is expressed as the resistance of the charge transfer between redox pairs occurring on the surface of the sample. The non-Faraday electrode capacitance owing to charge accumulation on the Fe_2O_3 surface is represented by a constant-phase element (CPE). Rp in parallel with the CPE is indicated by two charge paths for the electrons at the surface of the sample. The resistance Rs in series with both Rp and CPE represents the voltage drop in the electrolyte between the working electrode and reference electrode.

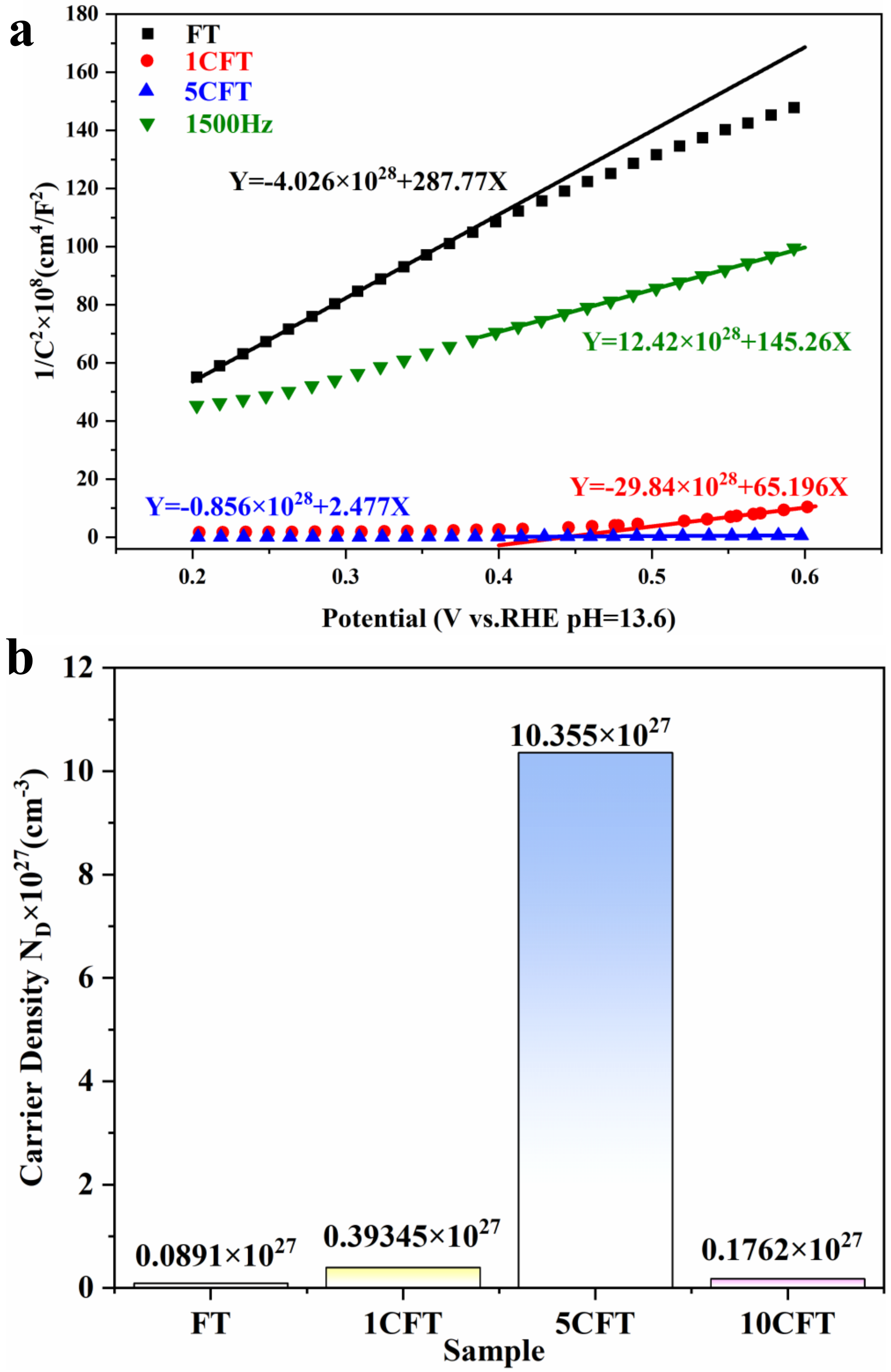

Figure S5. (a) Mott-Schottky plots collected at 1.5 kHz at a linear potential region between 0.2 to 0.6V *vs.* RHE and (b) Calculated carrier densities of Fe_2O_3 (FT) and Ce-doped Fe_2O_3 (1CFT, 5CFT, 10CFT).

References

[1] S. Kim, E. An, I. Oh, J.B. Hwang, S. Seo, Y. Jung, J.-C. Park, H. Choi, C.H. Choi, S. Lee, CeO_2 nanoarray decorated Ce-doped ZnO nanowire photoanode for efficient hydrogen production with glycerol as a sacrificial agent, ***Catalysis Science & Technology***, 12 (2022) 5517-5523.

[2] P. Senthilkumar, D.A. Jency, T. Kavinkumar, D. Dhayanithi, S. Dhanuskodi, M. Umadevi, S. Manivannan, N.V. Giridharan, V. Thiagarajan, M. Sriramkumar, K. Jothivenkatachalam, Built-in Electric Field Assisted Photocatalytic Dye Degradation and Photoelectrochemical Water Splitting of Ferroelectric Ce Doped $BaTiO_3$ Nanoassemblies, ***ACS Sustainable Chemistry & Engineering***, (2019).

[3] Y. Tan, S. Zhang, R. Shi, W. Wang, K. Liang, Visible light active $Ce/Ce_2O/CeO_2/TiO_2$ nanotube arrays for efficient hydrogen production by photoelectrochemical water splitting, International Journal of Hydrogen Energy, 41 (2016) 5437-5444.
